# Advancing global equity in cardiac care as cardiac implantable electronic device reuse comes of age

**DOI:** 10.1016/j.hroo.2022.08.006

**Published:** 2022-12-16

**Authors:** Daniel Alyesh, Behzad B. Pavri, William Choe, Mam Chandara, Mahmoud U. Sani, Phong Dinh Phan, Aime Bonny, Paul Khairy, Sunil K. Sinha, Uma Srivatsa, Joseph E. Marine, Kim Eagle, Thomas C. Crawford, Dhanunjaya Lakkireddy, Sri Sundaram

**Affiliations:** ∗ South Denver Cardiology Associates, Littleton, Colorado; † Thomas Jefferson University Hospital, Philadelphia, Pennsylvania; ‡ New World Heart Center, Phnom Penh, Cambodia; § Bayero University Kano & Aminu Kano Teaching Hospital, Kano State, Nigeria; ‖ Bac Mai Hospital, Hanoi, Vietnam; ¶ Gyneco-obstetric and Pediatric Douala Hospital, University of Douala, Douala, Cameroon; # Centre Hospitalier Le Raincy-Montfermeil, France; ∗∗ Montreal Heart Institute, Montreal, Canada; †† Johns Hopkins University School of Medicine, Baltimore, Maryland; ‡‡ University of California Davis Health, Sacramento, California; §§ University of Michigan, Ann Arbor, Michigan; ‖‖ Kansas City Heart Rhythm Institute, Overland Park, Kansas

**Keywords:** Pacemaker, Recycling, CIED, Low, Middle income countries

## Abstract

A nation’s health and economic development are inextricably and synergistically connected. Stark differences exist between wealthy and developing nations in the use of cardiac implantable electronic devices (CIEDs). Cardiovascular disease is now the leading cause of death in low- and middle-income countries (LMIC), with a significant burden from rhythm-related diseases. As science, technology, education, and regulatory frameworks have improved, CIED recycling for exportation and reuse in LMIC has become possible and primed for widespread adoption. In our manuscript, we outline the science and regulatory pathways regarding CIED reuse. We propose a pathway to advance this technology that includes creating a task force to establish standards for CIED reuse, leveraging professional organizations in areas of need to foster the professional skills for CIED reuse, collaborating with regulatory agencies to create more efficient regulatory expectations and bring the concept to scale, and establishing a global CIED reuse registry for quality assurance and future science.

**Key Findings**

▪ Cardiac implantable electronic device (CIED) recycling has the potential to make a significant impact on the leading cause of mortality in low- and middle-income countries (LMIC) worldwide.
▪ As science, technology, education, and regulatory framework have improved, CIED recycling for exportation and reuse in LMIC has become possible and primed for widespread adoption.
▪ CIED recycling is medically feasible, is ethical, and will have a positive economic downstream effect on developing nations.

A nation’s health and economic development are inextricably and synergistically connected.^1^ Stark health disparities exist between high-income countries and low/middle-income countries (LMIC), especially in resource-intensive specialties such as clinical cardiac electrophysiology (EP), cardiac implantable electronic device (CIED) utilization, and ablation of arrhythmias (Figure 1). For instance, in a high-income nation such as France, 782 pacemaker implants per million are performed annually, contrasted to 4 per million each year in a low-income country such as Pakistan.^2^ Poor global access to CIED therapy is estimated to account for at least 1 million deaths annually, as well as orders of magnitude more in physical and psychological morbidity and stunted economic activity.^3^^,^^4^

**Figure 1:**
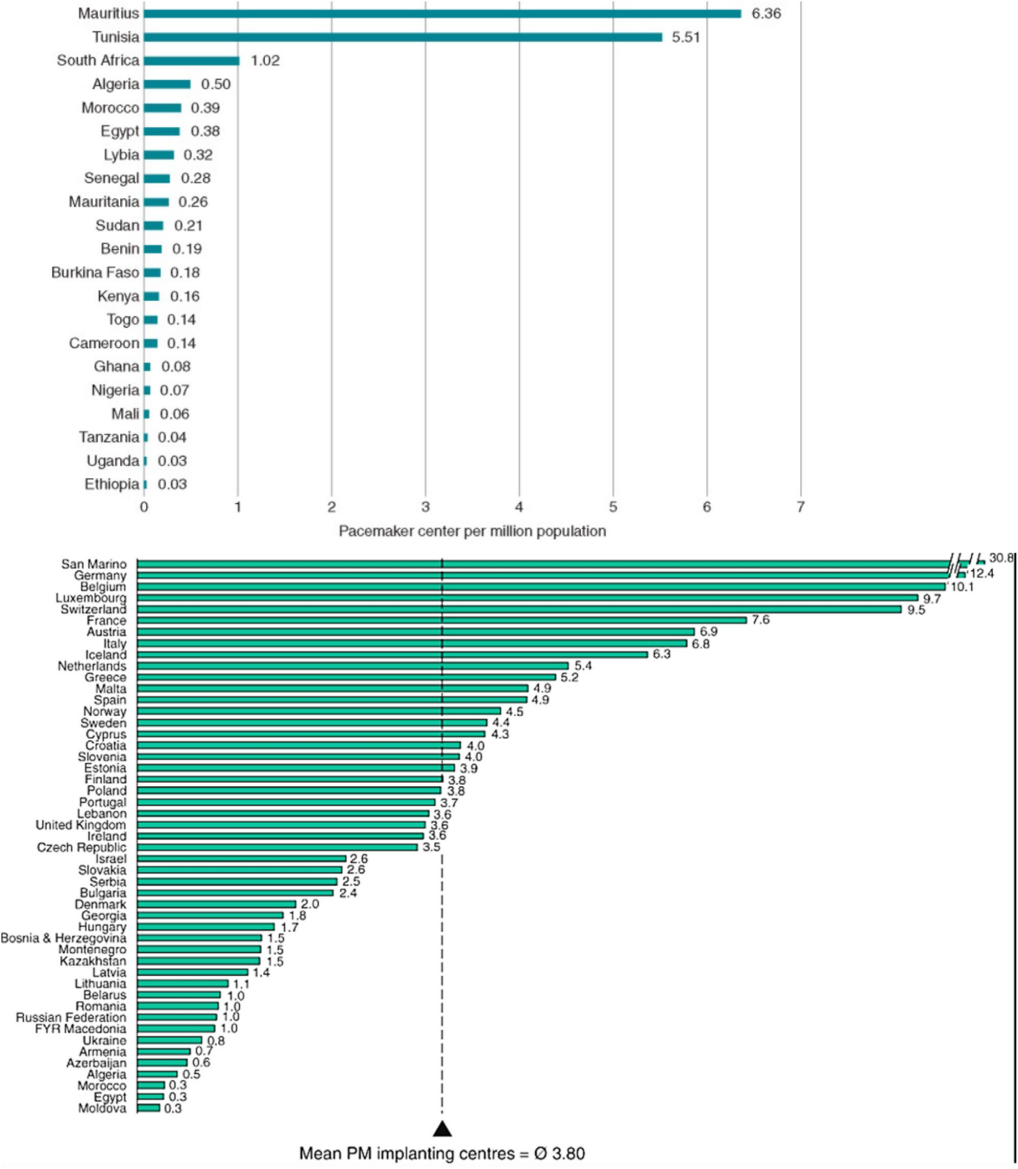
A comparison of the number of pacemaker implanting institutions per million inhabitants in European Society of Cardiology member countries (**A**) and Pan-African Society of Cardiology countries (**B**) in 2013.^5^^,^^6^

As science, education, and technology advance in the field of EP, this progress naturally transcends national and continental borders, albeit unevenly, with significant access disparities in LMIC. As a larger global EP community, it is our collective responsibility to target these disparities and identify concepts that may be ready for widespread adoption. No concept seems better positioned, tested, and primed for widespread adoption than CIED recycling for exportation and reuse in LMIC. Given the relative contributions of conduction disease vs sudden cardiac death, our discussion of CIED recycling will focus on pacing as a first step mirroring efforts and data to date. However, in time many of these concepts may be applied to broader CIED reuse. In this manuscript, we will characterize the need, review the evidence, and describe the regulatory framework around CIED recycling for export. In our experiences and based on the literature, the widespread practice of CIED reuse is hindered by conceptual misunderstanding and delays in learning to navigate the regulations rather than any inherent risks of the practice. The scientific data to support safe CIED reuse already exist and the regulatory requirements are already present, albeit complex. We believe that the broader EP community should recognize and address these issues as this concept “comes of age.” We have assembled broad and global perspectives to outline a way forward for CIED recycling for exportation to LMIC.

## The problem

While global public health efforts are often focused on communicable diseases such as HIV/AIDS, malaria, and other tropical diseases, noncommunicable diseases (NCDs) such as heart and lung disease and cancer now account for a larger share of global mortality (40.5 million in 2016) and cause the majority of deaths in LMIC. Cardiovascular diseases resulted in 17.9 million deaths in LMIC in 2016^7^; this is more than all reported deaths from the COVID-19 pandemic thus far.^7^

The burden of premature deaths owing to lack of access to CIED in cardiovascular diseases is profound, possibly underestimated, and a considerable societal burden in LMIC. In 2007, it was estimated that without a global effort to target chronic disease, approximately $87 billion in economic output would be lost from LMIC over a 9-year period.6 In developed nations, the average cost of implanting a pacemaker in the EP lab has been estimated at approximately $2700.^8^ To put this figure into context, the average annual gross national income per capita in an LMIC is between $1045 and $4095.^9^ Furthermore, in developing countries, patients requiring pacemakers or at risk for sudden cardiac death are younger than in high-income countries, leading to significant economic burden on their families and society.^10^^,^^11^ In an effort to combat this issue, the World Health Assembly adopted the World Health Organization’s Action Plan for the Prevention and Control of NCDs 2013-2020.^9^ In its plan, the WHO proposes a framework that empowers people and communities to take multisectorial action toward universal equitable coverage and innovative evidence-based strategies to enhance existing health solutions. As the guiding principles for this program, the WHO adopted approaches promoting life course, human rights, and equity, as well as international cooperation.

While it is difficult to precisely determine the relative contribution of a lack of CIED access to the increasing LMIC mortality statistics owing to cardiovascular diseases, it is likely significant. This is important context as the EP community advances CIED recycling to maturity. These efforts have the potential to reduce a significant portion of the leading cause of mortality in LMIC. Treating this cause of preventable premature death and disability may also have significant positive downstream effects on LMIC economic development.

## The history

The concept of pacemaker recycling originated in the 1970s and 1980s when a number of industrialized countries, including Canada, Australia, France, Sweden, and Norway, embraced pacemaker domestic generator reuse for domestic health care. Recognizing the potential economic benefits of such a practice, the North American Society of Pacing and Electrophysiology (now the Heart Rhythm Society [HRS]) in 1985 endorsed the concept of CIED recycling as long as it adhered to a rigorous process of testing, resterilization, informed consent, data analytics, and tracking.^12^ The tide against domestic CIED reuse began in 1980 when the United States Food and Drug Administration (FDA) published guidance against pacemaker reuse, citing concerns about the feasibility of device header resterilization.^13^ The European Union followed suit shortly thereafter, barring the practice.^12^^,^^14^ These regulatory barriers prevented any mainstream adoption of CIED recycling in the decades that followed.

In order for the concept of CIED recycling to regain acceptance, evidence generation and scientific validation was necessary. The cumulative scientific evidence has been analyzed in 2 large meta-analyses. In 2011, Baman and colleagues^15^ analyzed the early experience with pacemaker reuse in a large meta-analysis of studies dating back to 1980 that took place in both high-income and LMIC nations. Their analysis included 18 trials, 5 with control groups in a pooled analysis. They identified a low risk of infection of 1.97% and a low, yet notable risk of device malfunction of 0.68% in recycled devices.^15^ In 2018, Sinha and colleagues^16^ performed a meta-analysis of recent trial data for pacemaker and implantable cardioverter-defibrillator (ICD) reuse, including cardiac resynchronization therapy devices. The analysis included 9 observational studies, 5 with control groups, published between 2009 and 2017. All of the studies were performed in LMIC using comparable CIED screening interrogation, resterilization, and repackaging protocols using ethylene oxide gas. This analysis found that the rates of infection, device malfunction, and premature battery depletion were not significantly different from controls.^16^ A summary of trials included in these meta-analyses is shown in Table 1.

**Table 1:** Summary of trials included in cardiac implantable electronic device reuse meta-analyses

Study	Country	Year of completion	Number of reused pacemakers	Length	Complications related to device reuse	
					Infection	Failure
Balachander	India	1988	140	6 y	2	None
Pescariu et al	Romania	2001	365	35 ± 21 mo	6	None
Linde et al	Sweden	1996	100	32 ± 11 mo	2	1
Panja et al	India	1992	120	7.5 ± 5.6 y	6	None
Kruse	Sweden	1985	487		1	2
Kovacs	Hungary	1980	28		None	None
Cooperman	Israel	1984	78		None	None
Mond et al	Australia	1978	83		1	None
Amikam et al	Israel	1982	132	5 y	3	None
Havia et al	Sweden/Finland	1974	50	22 mo	1	None
Grendahl	Norway	1993	310		14	4
Costa et al	Brazil	1982	22	16 mo	1	2
Rosengarten et al	Canada	1987	18	29 mo	1	1
Sedney et al	Holland	1983	214	31.5 mo	1	1
Aren et al	Sweden	1979	19	26 mo	None	None
Ferugilo et al	Italy	1978	87	14 mo	1	None
Namboodri et al	India	2001	5	19.2 mo	None	None
Baman et al	Philippines	2008	12	4 mo	None	None
Hasan et al	Nicaragua	2011	17		None	None
Kantharia et al	India	2012	53		None	None
Pavri et al	India	2012	106		None	None
Nava et al	Mexico	2013	307		10	12
Feng et al	China	2014	99		3	1
Jama et al	South Africa	2015	63		0	1
Sosdean et al	Romania	2015	127		5	1
Selvaraj et al	India	2017	260		0	0

Most recently, Khairy and colleagues^17^ published the data from a multinational controlled prospective registry study further supporting the safety and efficacy of CIED reuse. This study analyzed the outcomes of recycled CIEDs (pacemakers, ICDs, and cardiac resynchronization therapy pacemakers and ICDs) implanted in LMIC in Latin America by experienced providers (>5 years’ experience) in comparison to a control group receiving new CIEDs in Canada. The study was a subset of a larger program that began in 1983 with the creation of a prospective registry in 2003. The results demonstrated a low 2-year infection rate (2.0% vs 1.2% in controls, *P* = .06) and no difference in device-related deaths.^17^

## A summary of programs to date

We applaud these programs and view these programs as foundational for scaling up of CIED reuse. Our source for this summary is from prior meta-analysis and members of our writing group who are active in ongoing CIED reuse efforts. All published programs we reviewed originated from single centers that are typically large and often have an academic component. In older studies, more developed nations were represented but newer data largely reflect the developing world.^15^ The oldest study originated in Sweden/Finland, where the process has largely been abandoned. The newest published study is out of a large academic center in Romania with ICDs procured from “Stimubanque,” an established organization with longitudinal experience in refurbishing CIEDs.^18^ Sterilization processes in these programs were largely consistent, although there appeared to be some variations: hydrogen peroxide vs alcohol-based sterilization agent, and use of additional biocidal agents such as iodine, varied.^16^ Of the trials presented, it is difficult to ascertain whether these programs tended more toward proof of concept vs ongoing sustainable programs. It seems a large share of the early programs in the developed world were halted, as regulatory agencies took an unfavorable view on the practice of CIED reuse. This is why increasing awareness and streamlining the regulatory environment around this practice is critical for scaling up of the process moving forward.

Robust programs that serve as proof of concept and are working to build capacity toward more widespread adoption of this technique include Project My Heart Your Heart (MHYH) at the University of Michigan (Ann Arbor, MI) and Heart Beat International out of the United Kingdom. These 2 programs have now combined their efforts. Heart Beat International serves as a resource for devices to be reconditioned in Michigan.^4^ MHYH has been working with the US FDA to develop a standardized exportation process that may be adopted by other programs. Additionally, MHYH has published a standardized sterilization process that may also be replicated.^19^ The program is now operating in 4 countries in a clinical trial and the organizers are working to add another 8 nations. The program employs a web-based registry that is easily accessed globally, with approximately 15 fields of data required for entry. This registry may be foundational for future global registry efforts, as it is easily adaptable to accommodate large data sets and many centers. The critique of MHYH is that its efforts are focused largely on laying the groundwork to demonstrate feasibility and efficacy. Larger efforts will need to move beyond this “proof of concept” phase.

## Behind the data toward standardized and adoptable processes

### Sources

The largest and most reproducible source of recyclable CIEDs appears to be from funeral homes and crematories, which represent the majority of published literature.^17^^,^^20^ Data published by the University of Michigan from a statewide effort with funeral homes revealed ≥21% of CIEDs had acceptable longevity, which was defined as ≥75% of remaining longevity or 4 years.^20^

Other possible sources are generators removed prematurely because of infection or system revision in the hospital and during postmortem examinations.^21^, ^22^, ^23^ Use of hospitals as sources of CIEDs would result in high yields of well-selected devices. About 50% of devices removed from patients at the University of Michigan EP laboratory for indications other than elective replacement indicator had the minimum of ≥75% of longevity or 4 years.^20^ FDA guidance issued in response to a number of device advisories in the early to mid 2000s recommends that explanted devices be returned to the manufacturer for analysis and product performance observations. This policy should be reconsidered in the light of alternative means of establishing device performance, such as remote monitoring and postexplant interrogation.

### Sterilization process

**Figure 2:**
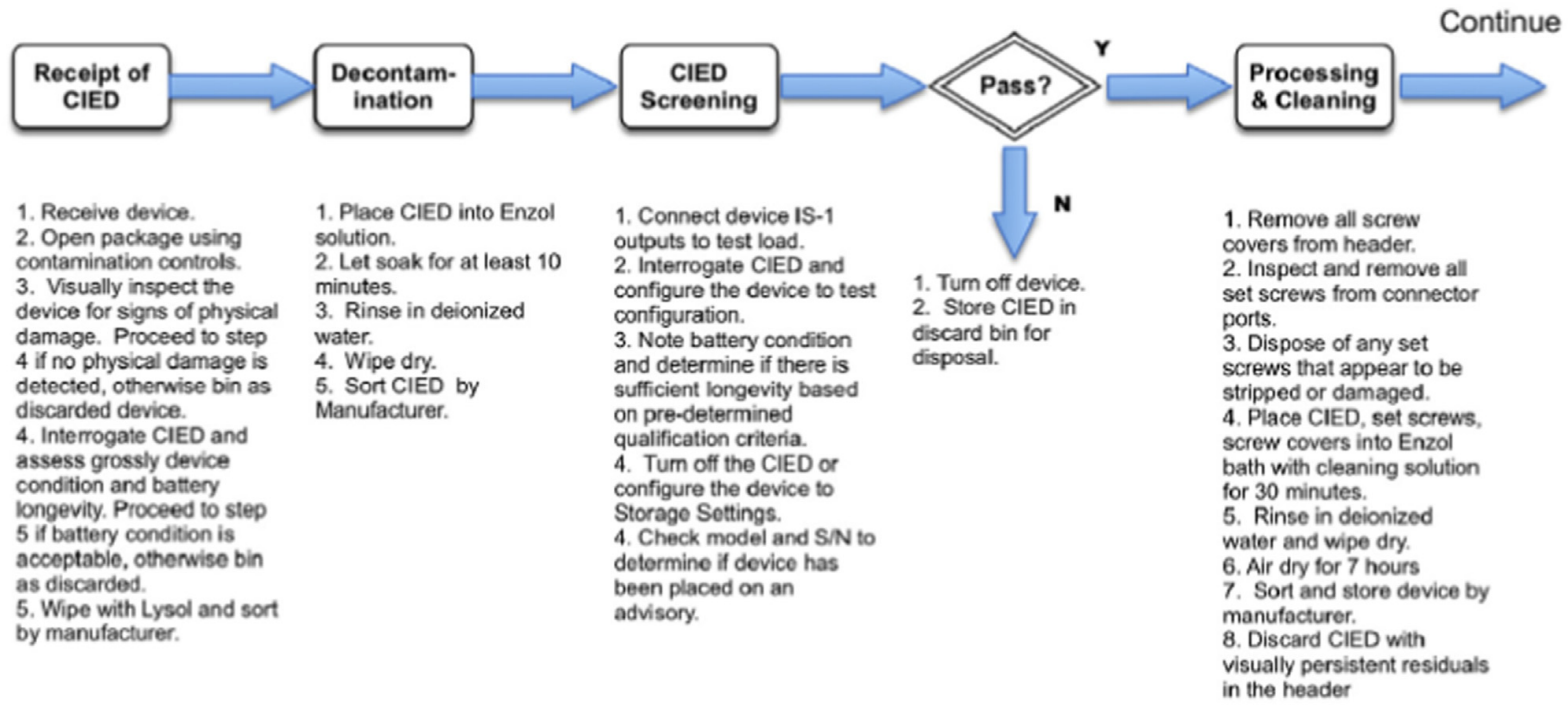
An illustration of the sterilization process in Project My Heart Your Heart (modified with permission from Journal of American College of Cardiology: Electrophysiology). CIED = cardiac implantable electronic device.

The potential for device infection owing to inadequate resterilization techniques seems to be the greatest source of concern from a regulatory and public acceptance standpoint. Data from the University of Michigan outlined a rigorous process that met medical industry standards and achieved a 12-log reduction of inoculated product and a sterility assurance of 10^-^^24^ while maintaining a high rate of appropriate device function.^19^ An illustration of this process is shown in Figure 2.

An important concept to address is a standardized approach to headers and set screws, which represented the FDA’s original objection to the concept of CIED recycling. A published report characterized in detail the process of screw removal, inspection, discarding of malfunctional or damaged apparatuses, and rigorous cleaning of those still acceptable for reuse.^17^^,^^19^ This protocol for reprocessing is the most data-driven approach and appears to be safe, at a modest cost per recycled CIED of $75–$100 (US dollars).^12^ However, a more conservative approach, as practiced in most of the studies included in the 2 aforementioned meta-analyses, may be to inspect and test all set screws and simply discard those CIEDs with set screws or grommets demonstrating damage or incomplete cleaning. This was also the practice in the large prospective registry study performed by Khairy and colleagues.^17^

Over the decades-long experience with the CIED sterilization process, ethylene oxide has been the method of choice for the final sterilization procedure. This process should adhere to ISO-11135—the standard established by the American National Standards Institute, Association for the Advancement of Medical Instrumentation, and International Organization for Standardization.^25^

### Leads and monitoring

The procurement of approved pacing leads for scaling up CIED reuse is crucial, since leads cannot be reused. A number of sources have been described in prior programs, ranging from manufacturer charitable donations to use of expired leads. However, growing this concept to scale likely cannot rely on these methods alone. The authors, many of whom are involved in ongoing programs, recommend approaching a manufacturer to supply leads at cost for CIED reuse programs, a model that has already been successful in existing smaller programs. Such resources are known to be available even in many recipient LMIC. For instance, because of previous efforts, many programs in sub-Saharan Africa have access to Medtronic leads and programmers. We recommend building on existing infrastructure in collaboration with recipient hospitals with CIED reuse efforts for better procurement of leads and to facilitate longitudinal follow-up based upon local infrastructure. This will provide for a sustainable and low-cost resource.

With regard to the device monitoring programs, we recommend a similar approach in LMIC to modern EP training programs: the physician must be trained not only to implant a device hardware but also to troubleshoot and manage its software longitudinally. All implanting institutions must have access to a device programmer, either as their own resource or from training institutions. Capacity for implantation must be built in combination with longitudinal in-person monitoring. This model already exists in MHYH. Through the HRS and other professional organizations, virtual coaching may also be made available by a group of volunteers, given broader and more seamless application of video-chat platforms. Remote monitoring is not a technology ready for this application in LMIC owing to inherent infrastructure issues, although it may be considered as programs mature.

## Ethical considerations

Historically, ethical concerns have been raised about recycled devices and their potential risks. We argue these concerns must be weighed against the ethics of not acting to prevent the significant morbidity and mortality related to poor CIED access in LMIC. Furthermore, the clinical evidence for CIED reuse demonstrates safety, and thus the ethical balance is in favor of pursuing this technology. One might argue that at the present time, withholding this technology can be viewed as unethical. We propose performing studies to understand the perceptions of CIED reuse in recipient countries. Our understanding of the ethical context cannot be complete without better understanding recipient perceptions. Furthermore, these perceptions are likely to vary across cultures and nations. Our diverse writing group includes perspectives from recipient nations to better represent this critical partnership.^26^ Based upon our understanding of the existing data, we propose that the informed consent process for recipients of CIEDs in LMIC include language about the resterilized device and our scientific understanding that there appears to be no significant increase in relative risk compared to a de novo device. While there remain many potential avenues for further inquiry about the ethics of CIED reuse, it is our general belief that the safety data combined with evidence of significant need in LMIC justify this effort as ethical, and one that should be widely adopted.

## Regulatory framework

The FDA objects to CIED domestic reuse on the grounds of infection risk and further classification as “an objectionable practice,” raising “a serious question whether pacemakers can be properly resterilized following initial implantation.”^13^ FDA approval of CIEDs for single use in the United States requires that manufacturers provide bench or clinical testing to demonstrate a “reasonable assurance of safety and effectiveness” without prior implant, use, refurbishment, recleaning, or resterilization. These recommendations date back to the year 2000, but the FDA’s domestic reuse policy is not the basis for CIED reuse for exportation. The FDA has collaborated with early adoption CIED reuse institutions to create a more transparent and defined process in a very important first step.

The FDA, in working with organizations advocating CIED reuse, has outlined a process for legally exporting devices to LMIC, as export of devices falls under a different regulatory context. This is a very important distinction. In brief, the process requires that the devices be characterized as not “contrary to public health, safety” by passing quality assurance, that they be labeled as “reprocessed not approved for use in the US,” and that a letter be obtained from an accepting foreign liaison from a recipient country communicating that the devices are being accepted as they do not conflict with the laws of the country and the recipient entity is aware of the status of the devices in the United States.^27^ This process has been quite effective, although obtaining such letters from recipient authorities can prove onerous for implanting physicians and hospitals.

## The way forward

Taken together, the emerging evidence base for CIED reuse, combined with the evolving regulatory framework around the practice, positions humanitarian CIED recycling as a concept primed for a “coming of age.” The global EP community’s perceptions of reuse are favorable, and while concerns regarding risk continue to be expressed, data have not supported an increase in risk.^15^, ^16^, ^17^^,^^28^ Similarly, surveys indicate that recycling of CIEDs after death or at the time of CIED removal are favorably viewed by most CIED “donor” patients.^29^ Finally, recently published data indicated that recipient patients and their family members support the concept of pacemaker reuse for patients who cannot afford new devices. The published evidence should change perceptions, and we as a community must now advance the culture around CIED recycling. Given the burden of cardiovascular disease in LMIC, and a likely significant contribution of conduction system disease in this cohort, the impact from widespread and robust application of this therapy could be profound. We propose the following next 4 steps:

### (1) Creating a task force to establish standards for CIED reuse

A group of key stakeholders in the global effort to assure high quality of reprocessed devices would have a sole focus to assure maximizing patient safety. Such a taskforce could be composed of delegates of the HRS and members of global specialty societies such as Asia Pacific HRS, Latin America HRS, Africa Heart Rhythm Association, and other relevant organizations, including national cardiac societies. Funding for such an effort may come from specialty societies or manufacturer charitable contributions. Establishing consensus standards for device procurement, cleaning, electrical testing, resterilization, implantation, and monitoring will be essential for assuring standardized levels of safety and efficacy. Centralizing the efforts around scaling this technology will also reduce duplicative efforts and better steward resources for individual practitioners and institutions. Currently, Project MHYH estimates the cost of sterilizing a device at $65 at a yearly volume of 250 devices a year. This is a commendable accomplishment, and economies of scale can further be realized with likely even lower costs as programs are expanded.

### (2) Leverage professional organizations in areas of need to foster the professional skills for CIED reuse

Enhancing the infrastructure for providing reused CIEDs to LMICs must be complemented by a corresponding focus on building the professional capability for device implantation and follow-up. A model of this concept may be found in the partnership between Project MHYH and the African Heart Rhythm Association and Pan-African Society of Cardiology. In recognizing the need for this expertise in Africa, the Pan-African Society of Cardiology has established a 6-month device implantation fellowship open to physicians in every sub-Saharan African country without a pacemaker implantation service, with a goal of addressing this deficiency by 2030.^30^^,^^31^ Future efforts must build on this model so that areas of need possess the skills to capitalize on the opportunity that CIED reuse presents.

### (3) Collaborate with regulatory agencies to create more efficient regulatory expectations to bring the concept to scale

Significant progress has been made through a partnership with FDA regulators, but this collaboration must meet the challenge of bringing CIED reuse for export to scale. While the current regulatory path was created with commendable effort, future endeavors must be streamlined. The newly formed task force should establish relationships with regulators to ensure safe and effective updated regulatory expectations in North America and Europe around CIED exportation for reuse. This process must require fewer resources, less protocol, and fewer steps to build on prior success.

### (4) Establishing a global CIED reuse registry for quality assurance and future science

There are many potential benefits of such a registry, including maintaining high-quality standards for CIED reuse and providing feedback to the reprocessors, clinicians, and regulators. The most recent study of CIED recycling employed a multinational prospective registry model developed at the Montreal Heart Institute and a similar scalable solution exists at University of Michigan MHYH. We propose an official registry be modeled in a similar manner to help categorize, study, and improve clinical practices. While we commend scientific efforts to date, the statistical power to understand and disseminate best practices, and to ensure quality control as CIED recycling accelerates in volume, would be more appropriate with a large registry, possibly in partnership with international EP professional societies.

In summary, the health of a nation and its economic activity are inextricably linked. CIED recycling, primed to take the next steps towards maturity, has the potential to make a significant impact on the leading cause of mortality in LMICs worldwide. Not only is it medically feasible and ethical, but the positive downstream effects on the economies of developing nations are as yet unmeasured and unrealized. We encourage the global EP community to take these next steps in advancing this international humanitarian practice in the years ahead.
